# Adding Concurrent Chemotherapy to Intensity-Modulated Radiotherapy Does Not Improve Treatment Outcomes for Stage II Nasopharyngeal Carcinoma: A Phase 2 Multicenter Clinical Trial

**DOI:** 10.3389/fonc.2020.01314

**Published:** 2020-08-07

**Authors:** Xiaodong Huang, Xiaozhong Chen, Chong Zhao, Jingbo Wang, Kai Wang, Lin Wang, Jingjing Miao, Caineng Cao, Ting Jin, Ye Zhang, Yuan Qu, Xuesong Chen, Qingfeng Liu, Shiping Zhang, Jianghu Zhang, Jingwei Luo, Jianping Xiao, Guozhen Xu, Li Gao, Junlin Yi

**Affiliations:** ^1^Department of Radiation Oncology, National Cancer Center/National Clinical Research Center for Cancer/Cancer Hospital, Chinese Academy of Medical Sciences and Peking Union Medical College, Beijing, China; ^2^Department of Radiation Oncology, Zhejiang Province Cancer Hospital, Hangzhou, China; ^3^State Key Laboratory of Oncology in South China, Department of Nasopharyngeal Carcinoma, Sun Yat-sen University Cancer Center, Collaborative Innovation Center for Cancer Medicine, Guangzhou, China

**Keywords:** nasopharyngeal carcinoma, intensity-modulated radiotherapy, concurrent chemoradiotherapy, stage II, treatment outcomes

## Abstract

**Purpose:** To explore the efficacy of concomitant chemotherapy in intensity-modulated radiotherapy (IMRT) to treat stage II nasopharyngeal carcinoma (NPC).

**Methods and Materials:** In this randomized phase 2 study [registered with ClinicalTrials.gov (NCT01187238)], eligible patients with stage II (2010 UICC/AJCC) NPC were randomly assigned to either IMRT alone (RT group) or IMRT combined with concurrent cisplatin (40 mg/m^2^, weekly) (CCRT group). The primary endpoint was overall survival (OS). The second endpoints included local failure-free survival (LFFS), regional failure-free survival (RFFS), disease-free survival (DFS), distant metastasis-free survival (DMFS), and acute toxicities.

**Results:** Between May 2010 to July 2012, 84 patients who met the criteria were randomized to the RT group (*n* = 43) or the CCRT group (*n* = 41). The median follow-up time was 75 months. The OS, LFFS, RFFS, DFS, and DMFS for the RT group and CCRT group were 100% vs. 94.0% (*p* = 0.25), 93.0% vs. 89.3% (*p* = 0.79), 97.7% vs. 95.1% (*p* = 0.54), 90.4% vs. 86.6% (*p* = 0.72), and 95.2% vs. 94.5% (*p* = 0.77), respectively. A total of 14 patients experienced disease failure, 7 patients in each group. The incidence of grade 2 to 4 leukopenia was higher in the CCRT group (*p* = 0.022). No significant differences in liver, renal, skin, or mucosal toxicity was observed between the two groups.

**Conclusion:** For patients with stage II NPC, concomitant chemotherapy with IMRT did not improve survival or disease control but had a detrimental effect on bone marrow function.

## Introduction

Nasopharyngeal carcinoma (NPC) has the highest incidence among head and neck cancers in Southeast Asia. Radiotherapy (RT) is the mainstay treatment modality for NPC. Concurrent chemoradiotherapy (CCRT), with or without adjuvant chemoradiotherapy, has been confirmed to have a significant survival benefit vs. RT alone for locally advanced NPC according to many prospective clinical trials and meta-analyses (1–6). Based on these studies, the NCCN guidelines have recommended CCRT with/without adjuvant chemotherapy as the standard treatment modality for patients with stage II–IVb (before the AJCC 8th edition) NPC since 2010 (7).

The benefit of concurrent chemoradiotherapy in locally advanced NPC is unquestionable. However, for stage II NPC, the role of concurrent chemotherapy remains unclear. The recommendation for concurrent chemoradiotherapy was based on only one phase 3 randomized trial published in 2011 by Chen et al. (8). In that trial, all patients were treated with two-dimensional radiation technique (2-DRT). Intensity-modulated radiotherapy (IMRT), which is characterized by advantageous dose distribution and reduced normal tissue exposure, has become to the mainstay radiation technique for NPC since the late 1990s. In the last two decades, the local control (LC) and overall survival (OS) of NPC have reached unprecedented levels with the use of IMRT, especially for patients with stage I/II disease, leading to almost 100% 3 year LC and OS, respectively (9). Therefore, it is rational to query whether any additional benefit can be introduced by the use of concurrent chemotherapy in stage II NPC treated with IMRT. To answer this, we conducted a multicenter phase 2 trial to assess whether concurrent chemotherapy could be omitted for patients with stage II NPC without compromising the overall treatment outcomes, yet avoiding the acute treatment-related toxicities associated with chemotherapy (1, 10–12).

## Patients and Methods

### Study Design

**Figure 1:**
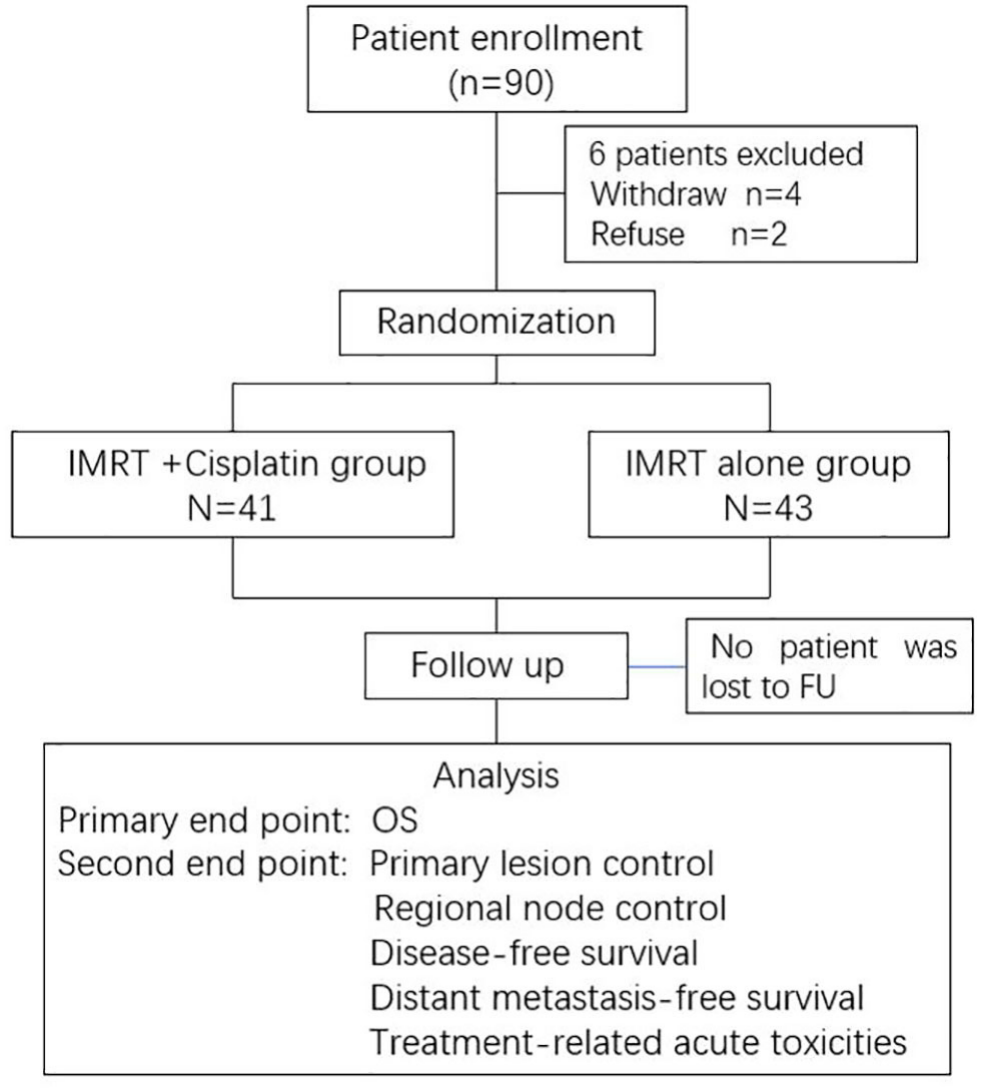
The CONSORT flow diagram.

This was a multicenter, randomized, phase 2 study. Eligible patients from three large cancer centers were registered and randomly assigned to receive either IMRT alone (IMRT group), or concurrent chemotherapy with IMRT (CCRT group). Patients were stratified according to the tumor (T) and node (N) classification using a central randomization method. The detailed study design is shown in a CONSORT flow diagram (Figure 1).

### Patient Eligibility

Eligible patients were required to have newly pathologically proven stage II NPC according to the 2010 UICC/AJCC staging system (T2N0, T1N1, or T2N1), a Karnofsky performance status (KPS) > 70, age ranging from 18 to 70 years, adequate hematological function (leukocyte count > 4 × 10^9^/L and platelet count >100 × 10^9^/L), normal renal function [serum creatinine level ≤ 1.25 × the upper limit of normal (ULN)], and normal hepatic function [alanine aminotransferase (ALT), aspartate transaminase (AST), and bilirubin (BIL) ≤ 1.25 × ULN]. Exclusion criteria included previous receipt of chemotherapy or radiotherapy, any other cancer history within 5 years, and any severe comorbidities that contraindicated the treatment in the procedure.

Before registration, all patients should receive the following workups: physical examination; endoscopy examination of the nasopharynx; magnetic resonance imaging (MRI) and computed tomography (CT) of nasopharynx and neck; chest CT; and abdominal and pelvic CT or ultrasound.

This study was approved by the Ethics Committee of the Cancer Hospital, Chinese Academy of Medical Sciences, and was registered with ClinicalTrials.gov (NCT01187238). Written informed consent was obtained from all patients before enrollment in the study.

### Treatment

All patients were treated using IMRT. Patients were immobilized using thermoplastic masks and simulated via a planning CT with 3 mm-thick slices. Intravenous contrast was strongly recommended. Target delineation was completed on the planning CT with the assistance of fused MRI images.

The gross tumor volume of the nasopharynx (GTVnx) was defined as the nasopharyngeal primary lesion displayed on simulation CT and diagnostic MRI. Cervical nodes with a short axis larger than 1 cm, with central necrosis, or a cluster of nodes large than 8 mm at level II, were considered positive and were named as GTVnd. The high-risk region of tumor invasion or nodal metastasis was defined as clinical tumor volume 1 (CTV1), including the entire nasopharynx, retropharyngeal nodal region, skull base, clivus, pterygopalatine fossa, parapharyngeal space, sphenoid sinus, and the posterior third of the nasal cavity/maxillary sinuses. CTV1 also included the regions with a high risk of nodal involvement, such as the level II nodal region for N0 patients or the corresponding level plus the adjacent level of positive nodes for N1 patients. Other nodal regions, including the supraclavicular fossa, were defined as CTV2. GTVnx, GTVnd, CTV1, and CTV2 were uniformly expanded by a 3-mm margin to generate the planning target volumes PGTVnx, PTV1, and PTV2, respectively.

Radiotherapy was delivered using simultaneous-integrated boost (SIB) IMRT, and all doses were prescribed to the PTVs. Generally, an RT dose of 69.96 Gy/2.12 Gy/33 fractions and 60.06 Gy/1.82 Gy/33 fractions were prescribed to the PGTVnx/GTVnd and PTV1, respectively. If there was a prophylactic neck volume (CTV2, prescribed dose was 50.96 Gy/1.82 Gy/28 fractions), the patients were treated using a two-phase plan. First, 28 fractions were delivered to all PTVs, and then the remaining five fractions were only delivered to PGTVnx/GTVnd and PTV1. If there were retropharyngeal lymph nodes with a diameter > 2 cm, the prescribe doses were 2.24–2.36 Gy/fraction for 33 fractions. The dose constraints for organs at risk were as follows: Maximum dose (Dmax) to 3 mm of the brain stem planning organ at risk volume (PRV): < 54 Gy; Dmax of 5 mm of the spinal cord PRV: < 45 Gy; Dmax of the optic nerve, chiasm, and temporal lobe: < 54 Gy; and the percentage of the volume receiving 30–35 Gy (V30–35) of the parotid gland was < 50%.

The patients randomized to the CCRT group also received concurrent chemotherapy of weekly cisplatin at 40 mg/m^2^, which was started on the first day of IMRT. A maximum of seven cycles of chemotherapy could be administered during radiotherapy.

### Follow-Up and Outcomes

All patients were followed up at 1 month after the completion of protocol treatment, every 3 months for the first 2 years and every 6 months for the 3rd to 5th years, and once a year thereafter. If there was suspicion of progression or toxicity, more frequent evaluations were allowed.

### Statistical Consideration

The primary endpoint of this study was overall survival (OS), which was defined as the period of time from the start of treatment to death from any cause. Secondary endpoints included local failure-free survival (LFFS), regional failure-free survival (RFFS), progression-free survival (PFS), distant metastasis-free survival (DMFS), and treatment-related acute toxicities. The National Cancer Institute Common Toxicity Criteria of adverse event (version 4.0) was used to assess treatment-related acute toxicities (13).

The SPSS 20.0 software (IBM Corp., Armonk, NY, USA) was used to analyze the data. The survival data were estimated using the Kaplan–Meier method, and the survival intervals of two groups were compared using the log-rank test. The chi-squared test was used to compare differences in acute toxicities and patient characteristics between two groups.

## Results

### Patient's Characteristics

Between May 2010 and July 2012, a total of 90 patients from three large cancer centers were screened. Six patients withdrew from the study after providing signed informed consent. Finally, 84 patients entered this study and completed the required treatment as per protocol, with 43 in the IMRT group and 41 in the CCRT group. The patients' general characteristics are listed in Table 1. The baseline characteristics were well-balanced between the groups. The median age was 48 and 46 years old for the CCRT and IMRT groups, respectively. There was no difference in terms of T and N stage between the two groups. All patients received the RT dose as per protocol, with a median of 70 Gy for both groups. With regard to the CCRT group, a median of 6 cycles of concurrent chemotherapy were completed, including 13 patients (31.7%) receiving 7 cycles, 20 patients (48.8%) receiving 6 cycles, 5 patients (12.2%) receiving 5 cycles, and the other 3 patients receiving ≤ 3 cycles of chemotherapy.

**Table 1:** The patients' characteristics between two groups.

**Items**	**CCRT (*****n*** **=** **41)**		**IMRT alone (*****n*** **=** **43)**		***P***
	***n***	**%**	***n***	**%**	
**Gender**					
Male	32	78.0	30	69.8	0.388
Female	9	22.0	13	30.2	
**Age**					
Median (Range)	48 (range 19–68)		46 (range 26–65)		
**T stage**					
T1	15	36.6	14	32.6	0.698
T2	26	63.4	29	67.4	
**N stage**					
N0	7	17.1	8	18.6	0.855
N1	34	82.9	35	81.4	
**Radiation Dose**					
Median (Range)	70 Gy (69.36–76.93 Gy)		70 Gy (69.7–74.25 Gy)		0.506

### Treatment Results

**Figure 2:**
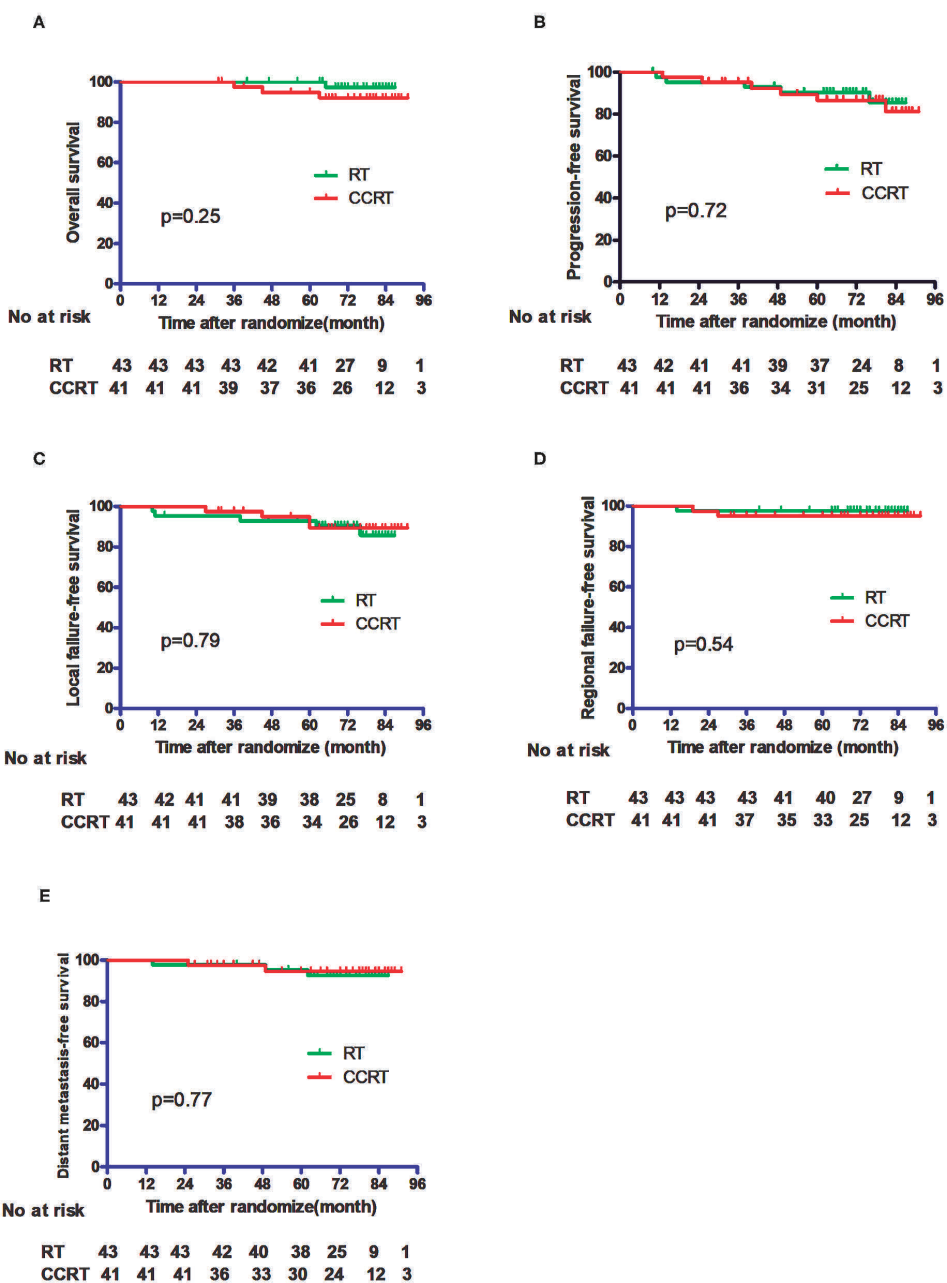
Comparison of the treatment results between the IMRT alone group and the CCRT group. **(A)** Overall survival. **(B)** Progression-free survival. **(C)** Local failure-free survival. **(D)** Regional failure-free survival. **(E)** Distant metastasis-free survival.

No patients were lost to follow-up. With a median follow-up time of 75 months, four patients died, including one from the IMRT group and three from the CCRT group. The 5 year OS, DFS, LFFS, RFFS, and DMFS for the whole cohort were 97.5, 88.7, 93.9, 96.4, and 94.9%, respectively. As shown in Figure 2, the OS, LFFS, RFFS, DFS, and DMFS of the IMRT and CCRT groups were 100% vs. 94.0% (*p* = 0.25), 93.0% vs. 89.3% (*p* = 0.79), 97.7% vs. 95.1% (*p* = 0.54), 90.4% vs. 86.6% (*p* = 0.72), and 95.2% vs. 94.5% (*p* = 0.77) respectively.

A total of 14 patients, 7 from each group, experienced treatment failure. There was no difference concerning the failure pattern between the two groups (Table 2). Five patients suffered distant metastasis with or without local–regional failure, including 4 patients with T2N1 and the other 1 with T2N0 diseases.

**Table 2:** Pattern of failure between IMRT and CCRT.

**Failure pattern**	**CCRT (*****n*** **=** **41)**		**IMRT alone (*****n*** **=** **43)**		***P***
	***n***	**%**	***n***	**%**	
Local	4	9.8	4	9.3	
Regional	1	2.4	0	0	
Distant Metastasis	2	4.8	1	2.3	
Local+Distant	0	0	1	2.3	
Regional+Distant	0	0	1	2.3	
Total	7	17.1	7	16.3	0.92

With regard to treatment-related adverse events, more grade 2–4 acute hematological (*p* = 0.02) and gastrointestinal (*p* = 0.02) toxicities were observed in the CCRT group than in the IMRT group. For hematological toxicity, a total of 5 patients presented ≥ G3 events in the entire study cohort. In the CCRT group, 3 grade 3 and 1 grade 4 events were observed whereas only 1 patient with grade 3 toxicity was reported in the IMRT alone group. A total of 5 patients experienced GI toxicities in the CCRT group, including 4 patients with grade 2 and 1 patient with grade 3 events. No ≥ G2 GI toxicity was observed in the IMRT group. There was no significant difference in terms of liver, renal, skin, and oral mucosa toxicities between the IMRT and CCRT groups (Table 3). No grade 3 xerostomia was observed in either group.

**Table 3:** Treatment related acute toxicities.

	**CCRT** ***n*** **=** **41**		**IMRT alone** ***n*** **=** **43**		***P***
	***n***	**%**	***n***	**%**	
Hb	1	24	0	0	0.3
WBC	17	41.4	8	18.6	0.02
PLT	1	2.4	0	0	0.30
GI	15	36.6	6	14.0	0.02
Liver	1	2.4	0	0	0.30
Skin	10	24.4	9	20.9	0.70
Mucositis	27	65.9	27	62.8	0.70

*Hb, hemoglobin; WBC, white blood cell; PLT, Platelet; GI, Gastrointestinal*.

## Discussion

Our study demonstrated that adding concurrent cisplatin to IMRT did not improve treatment outcomes in patients with stage II NPC but increased treatment-related acute hematological and gastrointestinal toxicities.

There are limited data assessing the role of concurrent chemoradiotherapy for stage II NPC. The only published phase 3 trial (8) that compared CCRT with RT alone found that the use of concurrent chemotherapy significantly improved the 5 year OS (94.5% vs. 85.8%; *p* = 0.007), PFS (87.9% vs. 77.8%; *p* = 0.017), and DMFS (94.8% vs. 83.9%; *p* = 0.007). However, it should be noted that all patients in that study underwent two-dimensional conventional radiotherapy, which has been proven to be inferior to IMRT. In addition, in Chen's study (8), 31 out of 236 patients (13.1%) had N2 disease (stage III) according to the 7th AJCC staging system. Therefore, the advantage of CCRT for this subgroup might have confounded the overall evaluation, leading to an overestimation of the role of concurrent chemotherapy for patients with pure stage II disease. It should also be noted that in that study, CCRT did not improve local regional control, with 5 year loco-regional relapse-free survival rates of 93.0% vs. 91.1% (*p* = 0.29), but did improve the distant metastasis-free survival, with the rates of 94.8% vs. 83.9%; *p* = 0.007), indicating that the decreased distant metastasis-free survival contributed to the improved OS.

In the present study, all patients were staged according to the 7th AJCC staging system with the assistance of MRI and CT imaging; therefore, the patients' tumor burden was more homogeneous compared with that of the abovementioned study. Additionally, the patients were pre-stratified by N status, leading to a minimized influence of N stage on the treatment results. Correspondingly, the 5 year DMFS of the CCRT and RT alone groups were 95.2% vs. 94.5% (*p* = 0.77), which were numerically higher than those reported in the previous study. Hence, the need for CCRT to decrease distant metastasis in our study was relatively unnecessary.

Although it has been widely confirmed by many randomized studies and meta-analyses that concurrent chemoradiotherapy could offer better treatment results than radiotherapy alone in locally advanced NPC, all of these studies showed that concurrent chemotherapy increased treatment-related toxicities, especially hematological, gastrointestinal, oral mucosal, and skin toxicities (1, 4, 10, 11). Our study also verified that CCRT increased treatment-related toxicities, even in patients treated using IMRT.

In the last two decades, IMRT has been widely used because of its advantage of dose distribution (14–16). Studies have confirmed that the advantage of dose distribution could translate into clinical benefit, in terms of either OS or treatment-related toxicities, especially for patients whose tumor was located in the center of the skull base and is surrounded by many critical organs (17). For patients with T1/T2 or stage I/II disease, Kwong et al. (9) reported the survival results of 33 patients with T1, N0–N1, and M0 NPC treated by IMRT and revealed that the 3 year LC, DMFS, and OS were all 100%. Several large sample studies from NPC epidemic regions reported 5 year OS rates of 80–85% in the IMRT era (18–23).

Stage II NPC has a relatively low tumor burden and a low risk of distant metastasis and indeed excellent LC, OS, and DMFS could be achieved using IMRT; therefore, doubts were expressed as to whether concurrent chemoradiotherapy is really needed in the era of IMRT. Fangzheng et al. (24) analyzed 242 patients with stage II disease treated by IMRT retrospectively and observed no significant differences between patients who received IMRT alone (*n* = 37), induction chemotherapy plus IMRT (*n* = 48), induction chemotherapy plus CCRT (*n* = 132), and CCRT (*n* = 25), with 5 year OS rates of 94.7, 98.7, 92.9, and 93.4%, respectively.

There have been few randomized studies focusing on the role of CCRT for stage II NPC treated by IMRT. Chen et al. (25) reported a randomized study with the same design as the present study and obtained similar findings. In Chen's study, 168 patients were recruited, of whom 160 were eligible for intent-to-treat analysis, with 81 in the CCRT group and 79 in the IMRT alone group. With a median follow-up of 61.5 months, the 5 year OS rates for the CCRT and IMRT alone groups were 91.4 and 88.6%, (*p* = 0.562). The 5 year DMFS rates were 93.82% in the CCRT arm and 93.67% in the IMRT alone arm (*p* = 0.967). There were significantly higher acute systemic side effects in the CCRT arm, especially the incidence of grade 3–4 hematological and gastrointestinal events (*p* = 0.000). Most of the locoregional recurrence (6/8, 75.0%) and distant metastases (6/7, 85.7%) occurred in the T2N1 group. Xu et al. (26) performed a systemic review and meta-analysis focusing on the value of chemoradiotherapy (CRT) in stage II NPC compared with that of RT alone. By including both 2D-RT and IMRT techniques, patients receiving CRT or RT alone achieved an equivalent OS, LRRFS, and DMFS (*p* = 0.14).

Considering that stage II consists of three subgroups (T1N1, T2N0, and T2N1), the prognosis and failure patterns might differ among these subgroups. In our study, a total of 5 patients experienced distant metastasis, including 4 harboring T2N1 tumor and the other 1 with T2N0 disease. Because of the relative small sample size and few events of distant metastasis in our study, it was not statistically meaningful for us to analyze the prognostic difference among the three subgroups. However, the T2N1 subgroup indeed accounted for the highest proportion of overall patients with distant failure. A series of publications provided retrospective evidence for this hypothesis. Leung et al. (27) found that patients with stage IIB disease had a higher distant failure rate when compared with patients in the stage I and stage IIA subgroups. Based on a database including 1,070 patients with NPC treated with RT alone from 1990 to 1998, Leung et al. (28) further reported a significantly higher isolated distant metastases rate (5.7% vs. 14.9%) for patients with T1–2N1 disease compared with that for the T2N0 subgroup. Similarly, Zong et al. (29) reported a 5 year accumulated distant metastasis rate of 10.8% in patients with T1–T2N1 disease vs. 0.1% in patients with T1–T2N0 NPC, accompanied by significantly different OS rates of 84.7% vs. 95.4% (*p* = 0.005). Xiao et al. (30) found that the accumulated distant metastasis-free survival rate was 81.2% for the T2N1 group, while the rates in the T1N1 and T2N0 groups were 95.6% and 97.5%, respectively, with corresponding 5 year OS rates of 73.1%, 95.6%, and 97.5%, respectively (*p* = 0.000). Even in Chen's (25) randomized study in which the design was similar to that of the present study, the T2N1 group demonstrated relatively worse outcomes compared with those of the other stage II subgroups, mainly because of increased failure in distant sites. The results of Chen's phase 3 study (8) confirmed that CCRT can decrease the distant metastasis rate for stage II NPC. Therefore, it would be important to distinguish patients with a higher risk of distant metastasis from general stage II patients to provide them with a more tailored treatment strategy. In recent decades, plasma-based Epstein–Barr virus DNA (EBV-DNA) evaluation has become an attractive prognostic biomarker. Leung et al. (27) observed that the probability of distant failure was significantly higher in patients with higher pretreatment plasma EBV-DNA levels (>4,000 copies/mL, *p* = 0.0001). Likewise, Du et al. (31) also verified that plasma EBV-DNA ≥ 4,000 copies/mL was independently associated with worse distant metastasis-free survival (DMFS) in 296 patients with stage II (AJCC 7th) NPC treated using IMRT.

In conclusion, this randomized phase 2 study demonstrated that adding concurrent chemotherapy to IMRT might not be necessary for stage II NPC. Considering the relatively small sample size and the implicit heterogeneity among patients with stage II disease, a further phase 3 study is warranted to confirm this finding in selected patients with stage II NPC with a lower risk of distant metastasis.

## Data Availability Statement

The datasets generated for this study are available on request to the corresponding author.

## Ethics Statement

This study was approved by the Ethics Committee of the cancer hospital, Chinese Academy of Medical Sciences, and was registered with ClinicalTrials.gov (NCT01187238). Written informed consent was obtained for all patients before enrollment to the study.

## Author Contributions

JY designed the study, wrote the protocol, reviewed all the case record forms for eligibility and protocol violation, recruited the patients, and wrote the manuscript. XiC, CZ, and LG designed the study. XH and JW were responsible for the patient accrual, radiotherapy treatment, data collection, and wrote the manuscript. LW, JM, CC, TJ, YZ, KW, YQ, XuC, QL, SZ, JZ, JL, JX, and GX were responsible for radiotherapy treatment, the patients' data collection, and data management. All authors contributed to the article and approved the submitted version.

## Conflict of Interest

The authors declare that the research was conducted in the absence of any commercial or financial relationships that could be construed as a potential conflict of interest. The handling editor declared a shared affiliation, though no other collaboration, with several of the authors CZ, LW, and JM.
